# The Influence Mechanism of Interfacial Characteristics between CSH and Montmorillonite on the Strength Properties of Cement-Stabilized Montmorillonite Soil

**DOI:** 10.3390/ma16227141

**Published:** 2023-11-13

**Authors:** Jinyu Ge, Fei Xu, Hua Wei, Qiang Wang, Hu Peng, Juan Zhou, Huaisen Li

**Affiliations:** 1 Materials & Structural Engineering Department, Nanjing Hydraulic Research Institute, Nanjing 210029, China; w8880600@163.com (J.G.); hwei@nhri.cn (H.W.); lihuaisen_nhri@163.com (H.L.); 2 Nanjing Highway Development Center, Nanjing 210008, China

**Keywords:** cement-stabilized montmorillonite soil, molecular dynamics method, interfacial characteristic, unconfined compressive strength, grey relational analysis

## Abstract

To elucidate the impact mechanism of the interfacial characteristics of Calcium Silicate Hydrate gel (CSH)–Montmorillonite (MMT) at the nanoscale on the strength of cement-stabilized montmorillonite soil, this paper begins by examining the interfacial energy. Through Molecular Dynamics (MD) simulation methods, the energy at the MMT and CSH binding interface is quantitatively calculated, and the correlation between the interfacial energy and macroscopic strength is determined in conjunction with grey relational analysis. Finally, based on the characterization results from X-ray diffraction (XRD), the accuracy and sources of deviation in the MD simulation results are discussed. The study shows the CSH-MMT interfacial energy is composed of van der Waals forces, hydrogen bond energy, and electrostatic interactions, which are influenced by the migration of cations; there is a good consistency between the CSH-MMT interfacial energy and the unconfined compressive strength (UCS) of cement-stabilized soil (cemented soil), with the interfacial energy decreasing as the number of water molecules increases and first decreasing then increasing as the number of MMT layers grows; by adjusting the mix proportions, the magnitude of the CSH-MMT interfacial energy can be altered, thereby optimizing the strength of the cemented soil.

## 1. Introduction

The implementation of ecological dredging is a crucial approach for the protection and management of the Yellow River basin, aimed at enhancing the quality and stability of its water ecosystem [1,2,3]. One key technical limitation hindering progress in dredging is the suboptimal utilization of mucky soil resources. Current research and practices are based on cemented soil technology, which improves the performance of mucky soil [4,5]. This improvement primarily involves adjusting the water-to-cement ratio (initial water content/cement content) and cement-to-soil ratio (cement content/soil content) to regulate mechanical properties such as the compressive strength [5,6,7].

The regulation effect of cemented soil’s performance is influenced by foundational properties like the soil’s liquid plastic limit [5,6], particle distribution [7], and organic matter content [8]. Liu et al. [6] introduced soil plasticity indices, particle distribution, and empirical parameters related to the reaction stages while coordinating with the water-to-cement ratio and cement-to-soil ratio to develop a reliable control model for the time evolution of cemented soil’s compressive strength. Although current control models for cemented soil strength [6,7] consider soil properties, the introduced empirical parameters lack physical meaning, making it difficult to reflect the interaction between cementitious materials and soil minerals at different ages. Additionally, current research on the development mechanism of cemented soil’s performance [9,10] primarily revolves around the hydration mechanism of cement and other cementitious materials, focusing on product and microstructure characterization. However, there is still a lack of analysis on the nanoscale interfacial binding mechanism of hydration products in cemented soil with clay minerals and the energy source of the cemented soil interface [11].

Molecular simulation techniques can effectively interpret interactions and energy changes between different phases on a nanoscale [12]. Scholars have successfully applied the Monte Carlo (MC) and MD methods to study the performance of inorganic non-metallic materials [13,14,15,16,17]. Nevertheless, considering the role of clay minerals, model construction and validation become more complex. Current studies mainly emphasize mechanical and structural evolution under varying conditions for single materials without thoroughly explaining the dynamic mechanisms behind the interactions between cement hydration products and soil minerals in cemented soil, leading to the mechanism behind the improved performance of cemented soil not being thoroughly explained.

Based on the analysis of the current research state, this paper first investigates the macro-mechanical performance mechanism of cement-stabilized montmorillonite soil influenced by a different water-to-cement ratio and cement-to-soil ratio through UCS tests. Subsequently, representative mineral components from cement hydration products and mucky soil, namely CSH and MMT, are selected to construct a composite nanoscale structure model. The evolution of non-bonded force fields between the nanoscale structure interfaces is computed to explore the interaction mechanism between CSH and MMT at a different water-to-cement ratio and cement-to-soil ratio, and how the characteristics of the CSH-MMT interface influence the macro properties of cement-stabilized montmorillonite soil. Later, the micro-phase is examined using XRD analysis. Combined with grey relational analysis, the accuracy and sources of deviation in the model are discussed, further verifying the MD model and the validity of its simulation results.

## 2. Materials and Methods

### 2.1. Experimental Materials

**Figure 1:**
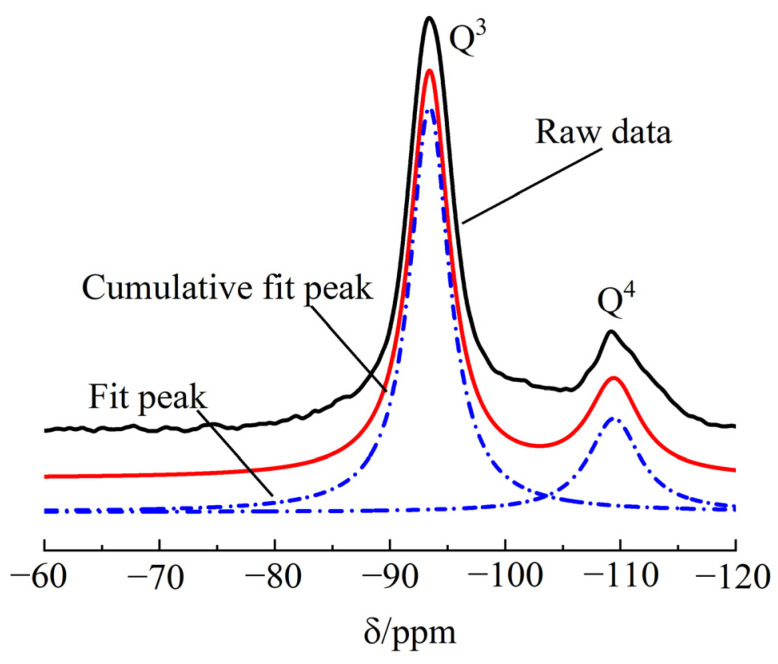
Deconvolution demonstration of ^29^Si NMR spectra for montmorillonite soil.

The soil used for both macroscopic and microscopic tests is sodium-based montmorillonite soil purchased from the market. Its specific gravity is 2.43. A peak-fitting method was applied to the soil’s ^29^Si nuclear magnetic resonance (^29^Si NMR) spectrum to determine its major mineral constituents and their concentrations [18], as shown in Figure 1. The results indicate that the soil consists of 67% montmorillonite and 33% quartz. The water used in the experiments is tap water from Nanjing, China. The cement used is Ordinary Portland cement 42.5 R. The physical properties of the cement are shown in Table 1, and CSH constitutes 60–70% of the hydration products of the cement.

### 2.2. Experimental Methods

#### 2.2.1. Molecular Simulation Method

This paper employs MD methods, using the open-source software Lammps (https://www.lammps.org/) to simulate the behavior of the CSH-MMT interface, with calculations performed using the Clayff force field. The Clayff force field can be used to calculate hydroxides and clay minerals and can simulate the properties of cement–clay systems and their interface with aqueous solutions [13,15,19]. The Clayff force field divides the total potential energy (*E*_total_) into bonded and non-bonded interactions [20]. Bonded interactions include bond stretching energy (*E*_bond stretch_) and bond angle bending energy (*E*_angle bond_), while non-bonded interactions include van der Waals forces (*E*_VDW_) and electrostatic interactions (*E*_Coul_), represented as in Equation (1).

*E*_total_*= E*_bond stretch_*+ E*_angle bond_*+ E*_VDW_*+ E*_Coul_ (1)

Electrostatic interactions and van der Waals forces are defined as in Equations (2) and (3), respectively, where (*e*) is the electronic charge; (*ε*) is the potential well depth; (*σ*) is the atomic distance where the potential energy is at its minimum; (*q_i_*) and (*q_j_*) are the charges of atoms (*i*) and (*j*); and (*ε*_0_) is the permittivity constant. The distance parameter (*σ_ij_*) is the arithmetic mean of (*σ_i_*) and (*σ_j_*), and the energy parameter (*ɛ_ij_*) is the geometric mean of (*ε_i_*) and (*ε_j_*).

(2) $$E_{\mathrm{Coul}}=\frac{e^{2}q_{i}q_{j}}{4\pi \varepsilon_{0}r_{ij}}$$

(3) $$E_{\mathrm{VDW}}=\varepsilon_{ij}\left[{\left(\frac{\sigma_{ij}}{r_{ij}}\right)}^{12}-2{\left(\frac{\sigma_{ij}}{r_{ij}}\right)}^{6}\right]$$

#### 2.2.2. UCS Testing and Sample Preparation

UCS testing is employed to assess the mechanical properties of the specimens. According to engineering experience, the content of cementitious materials in the cemented soil should range from 7% to 25% of the soil’s weight. Within this range, variations in the water-to-cement ratio and the cement-to-soil ratio are investigated to explore their influence on the UCS of the cemented soil. Ultimately, the following mix proportions are determined: when the water-to-cement ratio is 4, the cement-to-soil ratio is set at 7.5%, 15%, 20%, and 25%. Conversely, when the cement-to-soil ratio is 25%, the water-to-cement ratio is adjusted to 1.33, 2, 3, and 4. Samples are named in the format of “water-to-cement ratio–cement-to-soil ratio”, such as 4–25 for cemented soil with a water-to-cement ratio of 4 and a cement-to-soil ratio of 25%.

Samples of cemented soil with a size of *ϕ* 50 mm × 50 mm and a porosity of 3% are prepared using the absolute volume method. This ensures that, under the same mix proportions, the likelihood of contact reactions between particles inside the samples is consistent at the start of the reaction. UCS tests selected the samples with a reaction age of 90 d. The testing equipment employed is the HY-10080 electronic universal material testing machine from Shanghai Hengyi, Shanghai, China, with a loading rate of 1 mm min^−1^. The results are based on the average of three parallel samples.

#### 2.2.3. XRD

XRD is used for the qualitative analysis of the mineral composition of cemented soil and to validate and supplement the results obtained through molecular simulation. For samples aged to the predetermined age, hydration is terminated using freeze-drying [21], and the samples are ground to a particle size not exceeding 75 μm for XRD testing. The testing equipment used is the SmartLab-9Kw diffractometer from Rigaku, Tokyo, Japan, employing Cu target Kα radiation, with a tube voltage of 40 kV, tube current of 30 mA, a scanning rate of 2°/min, a scanning range of 5° to 60°, and a step size of 0.02°.

### 2.3. Establishment and Rational Validation of MD Models

#### 2.3.1. Construction and Verification of Typical CSH and MMT MD Models

**Figure 2:**
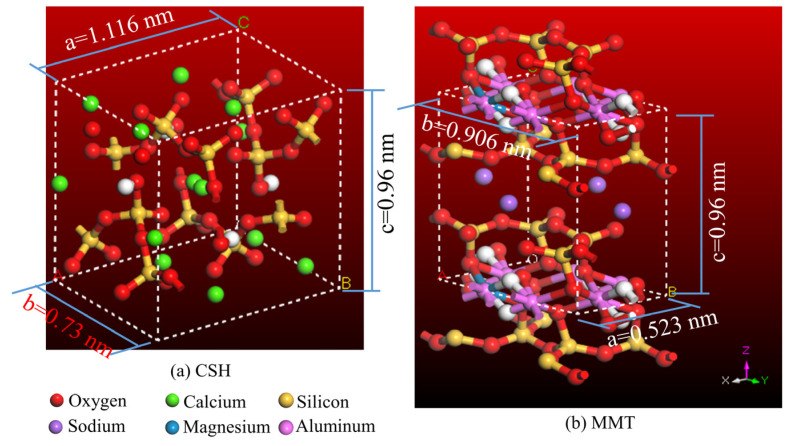
CSH and MMT models.

CSH is a gel with an atomic structure resembling that of layered tobermorite. Generally, depending on the degree of hydration and interlayer spacing, CSH models can be represented by Tobermorite—0.9 nm, Tobermorite—1.1 nm, and Tobermorite—1.4 nm, as reported in references [22,23]. Among these, the Tobermorite—0.9 nm structure does not contain water molecules, while the Tobermorite—1.1 nm and Tobermorite—1.4 nm structures contain 5 and 7 water molecules, respectively. To reflect the nanoscale structural interfacial performance of hydrated cement-stabilized montmorillonite soil pastes under varying moisture contents, and considering MMT’s strong affinity for water molecules, Ca^2+^, and CaOH^+^, a CSH model based on the Tobermorite—0.9 nm structure with low Ca/Si and interlayer water content is constructed. The structure is shown in Figure 2a, with lattice parameters: a = 1.116 nm, b = 0.73 nm, c = 0.96 nm, α = 101.08°, β = 92.83°, γ = 89.98°.

MMT is a typical layered aluminosilicate mineral composed of two layers of silicon–oxygen tetrahedra and a layer of aluminum–oxygen octahedra sandwiched in between. The lattice parameters for the MMT model, as proposed by Skipper [24], are a = 0.523 nm, b = 0.906 nm, c = 0.96 nm, α = γ = 90°, β = 99°. Figure 2b depicts the structure model of MMT, where every 32 Si^4+^ are substituted by Al^3+^, and every 8 Al^3+^ are substituted by Mg^2+^. Consequently, due to lattice substitutions, the MMT layers exhibit electronegativity, balanced by interlayer Na^+^. The formulated molecular formula for MMT is Na_3_(Si_31_Al)(Al_14_Mg_2_)O_80_(OH)_16_.

To validate the correctness of the constructed MD models, the Young’s modulus (E) of CSH and MMT is studied. The computed results are compared with experimental data from other literature sources [25,26,27]. Prior to formal calculations, energy and geometry optimization of the structures is carried out to reach the lowest potential energy configuration. Subsequently, to ensure both temperature and energy equilibrium in the structural system, 300 ps of dynamic optimization is performed successively under NVT and NVE ensembles. Finally, to maintain structural elasticity during the calculation process, the constant-strain method is utilized to compute the elastic constants with a maximum strain set at 0.003.

Analysis of stiffness and compliance matrices in the computed results reveals that although CSH and MMT are not isotropic materials, they are not highly anisotropic either. For simplicity in calculations, these structures are approximated as isotropic materials, and the Voigt–Reuss–Hill method is employed to compute the bulk and shear moduli. Ultimately, Young’s moduli for CSH and MMT are determined to be 34.39 GPa and 50.05 GPa, respectively, using Equation (4).

(4) $$E=\frac{9G}{3+G/K}$$

In the formula, (G) represents the shear modulus and (K) represents the bulk modulus. Du et al. [25] experimentally determined the Young’s modulus of CSH to be in the range of 30 GPa to 50 GPa. The molecular simulation result obtained in this study, 38 GPa, aligns well with these findings. Carrier et al. [26], through MD simulations, obtained an elastic modulus of 34.2 GPa for sodium-based MMT at room temperature, which is lower than the 50.05 GPa obtained in this study. This discrepancy can be attributed, in part, to the fact that the Young’s modulus of MMT crystals decreases as the number of interlayer water molecules increases. Additionally, differences in the simulation conditions, temperature, pressure, and force fields can lead to variations in the simulation results. Wang et al. [27], using elastic wave testing on MMT powder, reported a Young’s modulus slightly higher than our computed values, ranging from 41.3 GPa to 60.6 GPa. This difference is mainly due to the fact that elastic parameters measured through acoustic or elastic wave methods generally tend to be higher than static measurements. Based on the above analysis, the numerical values obtained through simulation align well with experimental data from the literature, which to a certain extent, supports the correctness of our model and mechanical processing methods.

#### 2.3.2. Construction of the CSH-MMT Interfacial Model

**Figure 3:**
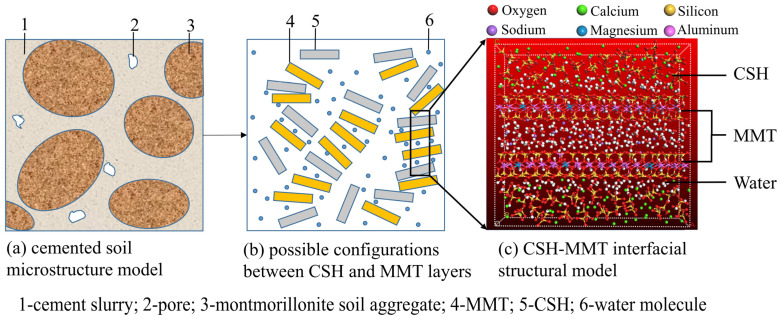
Illustration of CSH-MMT from microscopic to molecular structure.

Figure 3 is an illustration of CSH-MMT from the microscopic to molecular structure. After thorough mixing of cement and montmorillonite soil, cement particles, montmorillonite soil particles, and pores are uniformly distributed within the structure. With the addition of water, montmorillonite soil particles aggregate into larger soil aggregates. Cement hydrates on the surface of montmorillonite soil aggregates to form a cement slurry. Due to the weak interlayer bonding of MMT, water molecules can penetrate the interlayers, causing strong expansion. This process compresses and fills the partial pores within the soil aggregates, resulting in a dense cement–montmorillonite soil structure, as depicted in Figure 3a. Given the dispersed-layer structure of the cement–montmorillonite soil microstructure, and considering possible configurations between CSH and MMT layers (Figure 3b), the Build Layers method is employed to construct the CSH-MMT interfacial structural model. The model is assembled in three layers, namely, the upper CSH chain layer, the middle MMT crystal layer, and the lower CSH chain layer, as shown in Figure 3c.

To ensure consistency between the molecular model and the mix proportion, the model construction controls the number of water molecules and the number of MMT layers. This approach achieves an approximate equivalence between the masses of CSH, MMT, and water in the molecular model and macroscopic experiments. Specifically, the molecular model maintains a constant of 2 CSH layers and 160 water molecules, while varying the number of MMT layers as 2, 3, 4, and 6. These correspond to macroscopic experiments with a water-to-cement ratio of 4 and cement-to-soil ratio of 7.5%, 15%, 20%, and 25%. The molecular model also maintains 2 CSH and MMT layers, while varying the number of water molecules as 160, 120, 80, and 50. These correspond to macroscopic experiments with a cement-to-soil ratio of 25% and water-to-cement ratio of 1.33, 2, 3, and 4.

**Figure 4:**
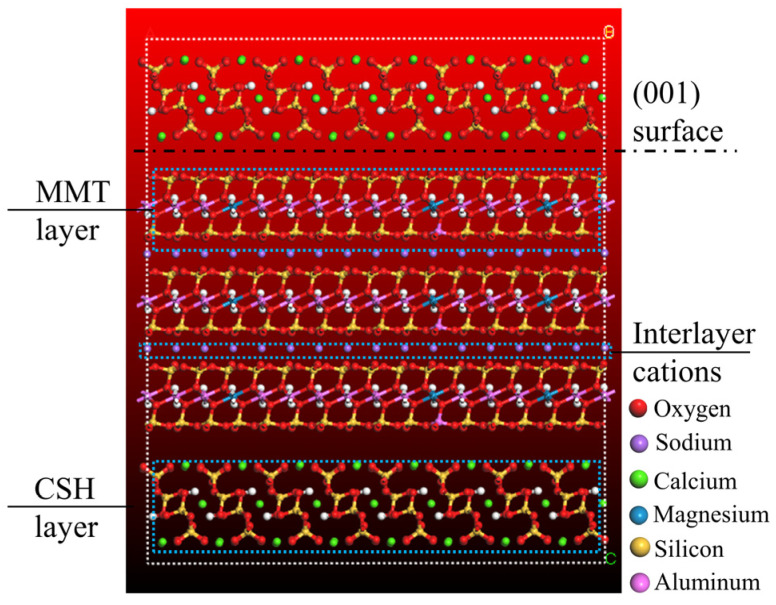
CSH-MMT interfacial model with 3 MMT layers.

The specific construction process involves first performing supercell operations on the intermediate MMT unit cell to create supercell structures containing 2, 3, 4, and 6 layers of MMT. Subsequently, the (001) surfaces of both CSH and MMT supercells are extracted, and the structure is assembled in three layers using the Build Layers method. A structure model with 3 layers of MMT is depicted in Figure 4, with a dashed line representing the interaction surface (001) between CSH and MMT. After establishing the CSH-MMT interfacial structural models, the MC method is used to simulate the water absorption process. A total of 160 water molecules are added to the structural models containing 2, 3, 4, and 6 layers of MMT, and 160, 120, 80, and 50 water molecules are added to the structural model containing 2 layers of MMT, yielding molecular models of the CSH-MMT-water system.

## 3. Results

### 3.1. CSH-MMT Interfacial Energy

#### 3.1.1. MD Calculation of the Interface between CSH and MMT

Upon obtaining the molecular model of the CSH-MMT-water system, energy and geometric optimization are performed. Initially, the structures are optimized using the steepest descent and conjugate gradient methods to find configurations near the energy minimum on the potential energy surface. Subsequently, the Newton–Raphson method is employed to further optimize the structures and locate the configurations at the minimum of the potential energy surface, reducing unreasonable arrangements between molecules. The charge distribution employs the QEq method, which assigns charges to each atom, allowing for the fast computation of the electrostatics within the periodic framework. The energy-minimized model resulting from relaxation is used as the initial model for the MD simulations. The boundary conditions for the MD simulations are set to three-dimensional periodic boundary conditions, using the NPT ensemble to maintain a constant particle number, pressure, and temperature. The precision is set to “fine”. Additionally, temperature and pressure control for the water-CSH-MMT system is implemented using the Nose–Hoover thermostat and Berendsen barostat, with temperature and pressure constants set to 0.5 fs. The system is then subjected to 300 ps of dynamic equilibrium to observe energy and temperature convergence over time, reaching a state of equilibrium. Finally, the total energy of the CSH-MMT-water system, the energy of CSH, and the energy of the MMT-water system after removing CSH are calculated using the energy method. Specifically, the energy of the equilibrium configuration is calculated to obtain the total energy (*E*_total_). Then, CSH layers are removed from the equilibrium configuration, and the energy of the MMT-water system without CSH (*E*_MMT/water_) is computed. Subsequently, calculations are performed with only the CSH layers to obtain the energy of pure CSH (*E*_CSH_). Finally, the interfacial energy is determined using Equation (5). The Ewald summation method is employed to calculate the static electrostatic interactions within the structure, with a cutoff radius of 0.6 nm, while the atom-based method is used to compute van der Waals forces, with a cutoff radius of 1.25 nm. The choice of cutoff radius is based on being less than half of the crystal parameters.

#### 3.1.2. CSH-MMT Interfacial Energy

This study analyzes the interfacial properties of CSH-MMT from the perspective of interfacial energy. The formula for calculating the interfacial energy (*E*_interface_) is as shown in Equation (5). Since all models have the same interfacial area (A), Equation (5) can be simplified to Equation (6):

(5) $$E_{\mathrm{interface}}=\frac{E_{\mathrm{CSH}}+E_{\mathrm{MMT}/\mathrm{water}}-E_{\mathrm{total}}}{2A}$$

(6) $$E_{\mathrm{interface}}=E_{\mathrm{CSH}}+E_{\mathrm{MMT}/\mathrm{water}}-E_{\mathrm{total}}$$

**Figure 5:**
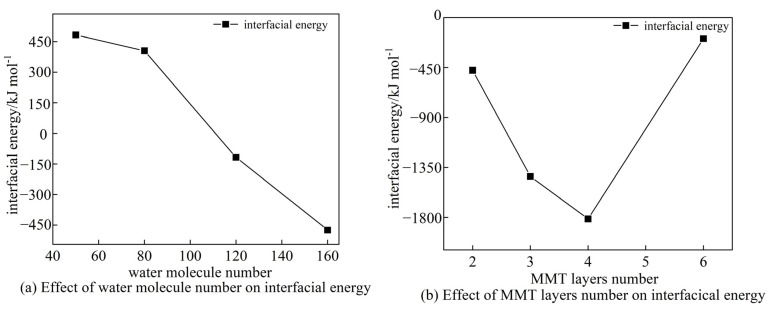
CSH-MMT interfacial energy.

Figure 5a,b depict the interfacial energies of CSH-MMT with varying numbers of MMT layers and water molecules, respectively. A positive interfacial energy indicates a repulsive force between adjacent surfaces, while a negative interfacial energy indicates an attractive force.

**Figure 6:**
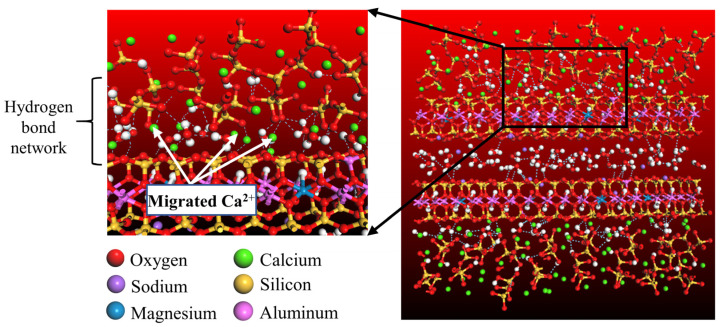
Schematic diagram of the hydrogen bond network at the CSH-MMT interface.

The interfacial energy is primarily composed of van der Waals forces, hydrogen bond energies, and electrostatic interactions. Van der Waals forces can either be attractive or repulsive, depending on the distance between atoms: they are attractive at greater distances and repulsive at close distances. Hydrogen bond energy is a significant contributor to the attractive force at the CSH-MMT interface. The strength of the hydrogen bond network at the CSH-MMT interface determines its magnitude. This network forms due to the following reasons: as mentioned earlier, MMT exhibits significant electronegativity due to lattice substitution, attracting Ca^2+^ from CSH into the interlayer space. These Ca^2+^ bond with oxygen atoms (O_s_) on the surface of CSH and MMT, as well as diffusing to coordinate with water molecules’ oxygen atoms (O_w_) in the CSH-MMT interlayer space, forming Ca-O_s_ and Ca-O_w_ bonds. Simultaneously, water molecules bond via H_w_ with O_s_ and O_w_, forming O_s_-H_w_ and O_w_-H_w_ bonds, where O_s_ is from the surfaces of CSH and MMT, and O_w_ is from other water molecules. Through these series of interactions, a complex hydrogen bond network is established at the CSH-MMT interface, as shown in Figure 6. Electrostatic interactions follow the principle of like charges repelling and opposite charges attracting. Cations migrate between layers, leading to electronegative MMT attracting free Ca^2+^ from CSH. This causes CSH to develop a positive charge, and as MMT layers are negatively charged, electrostatic forces result in mutual repulsion between the CSH and MMT layers.

From Figure 5a, it can be observed that, when the number of water molecules decreases while maintaining the same number of CSH layers and MMT layers, the interfacial energy increases. The interfacial energy reaches its maximum of 482.8 kJ mol^−1^ when the number of water molecules is reduced to 50. This situation occurs because, as the number of water molecules decreases, Ca^2+^ begin to migrate toward the interface region due to the attractive forces exerted by the MMT layers. This causes both the CSH and MMT layers to become positively charged, leading to mutual electrostatic repulsion and an increase in the interfacial energy. Notably, when the number of water molecules decreases from 80 to 50, the interfacial energy only increases by 77.4 kJ mol^−1^. This phenomenon can be attributed to the fact that the adsorption of Na^+^ by the MMT layers reduces their zeta potential, diminishing the significance of electrostatic interactions in the system, which is why the increase in the interfacial energy is less pronounced than the preceding significant decrease. When the number of water molecules increases from 50 to 160, the interfacial energy transitions from repulsive to attractive. This change is due to the increased spacing between the CSH and MMT layers at the interface as the number of water molecules rises, resulting in van der Waals forces predominantly exhibiting attraction. Additionally, more O_w_-H_w_ bonds form in the CSH-MMT interface region, further increasing the attractive forces. Moreover, the electronegativity of the MMT layers becomes balanced by water molecules, reducing the electrostatic interactions. Figure 5b shows that, with a constant number of water molecules, the interfacial energy initially decreases and then increases as the number of MMT layers rises. As previously mentioned, this outcome is closely related to the charged nature of the CSH layers in the system. With an increasing MMT content, free Na^+^ migrate into the CSH, forming a new gel C-N-S-H [28]. This results in a positively charged CSH layer. Due to the negatively charged MMT layers, electrostatic forces cause mutual attraction between the CSH and MMT layers. Additionally, Figure 5b demonstrates that when the number of MMT layers continues to increase to six, the interfacial energy significantly increases. The analysis reveals that, at this point, the number of interlayer water molecules is relatively low, the hydrogen bond energy is a small fraction of the total, and Ca^2+^ in the CSH are once again attracted by the MMT layers. As a result, electrostatic interactions in this system lead to repulsion and, consequently, an increase in the interfacial energy.

### 3.2. UCS and Its Correlation with CSH-MMT Interfacial Energy

**Figure 7:**
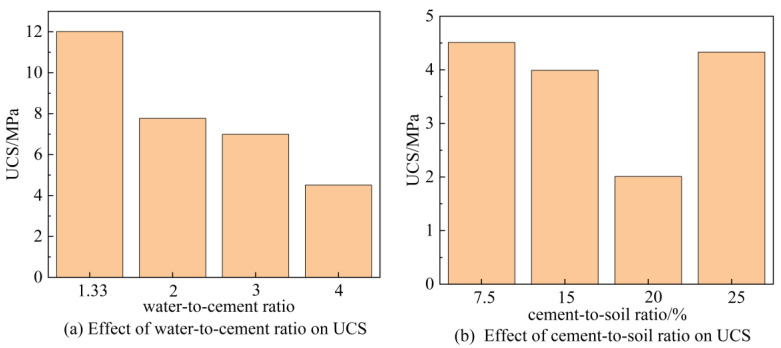
UCS test results of cement-stabilized montmorillonite soil.

The UCS test results are presented in Figure 7. From Figure 7, it can be observed that the UCS of cemented soil decreases with the increase in the water-to-cement ratio when the cement content is kept at 25%. When the water-to-cement ratio remains unchanged at 4, with the increase in the cement content from 7.5% to 20%, the UCS of cemented soil decreases from 4.51 MPa to 2.01 MPa. However, as the cement content further increases to 25%, the UCS of the cemented soil increases by 115%.

One of the aims of this study is to investigate whether the CSH-MMT interfacial characteristics affect the strength of cement-stabilized montmorillonite soil. Therefore, the present section uses grey relational analysis to determine the degree of importance of the CSH-MMT interfacial energy on the development of macroscopic properties in cement-stabilized montmorillonite soil. If the degree of importance is high, it indicates that the energy in the interface binding region between CSH and MMT is a crucial factor influencing macroscopic performance, and we can further explore the mechanism by which the CSH-MMT interfacial energy affects the strength of cement-stabilized montmorillonite soil.

We can use the calculated CSH-MMT interfacial energy from the MD simulations as a reference sequence and the UCS of cement-stabilized montmorillonite soil as a comparative sequence, which are recorded as Equation (7) and Equation (8), respectively, where (*n*) is the length of the sequence.

(7) $$x_{i}^{\prime }=(x_{i}^{\prime }\left(1\right),x_{i}^{\prime }\left(2\right),\cdots x_{i}^{\prime }\left(n\right))$$

(8) $$x_{0}^{\prime }=(x_{0}^{\prime }\left(1\right),x_{0}^{\prime }\left(2\right),\cdots x_{0}^{\prime }\left(n\right))$$

Grey relational analysis is calculated according to the method described in reference [17], and the specific steps are as follows: Firstly, the reference sequence and the comparison sequence are dimensionless processed according to Equation (9) and Equation (10), respectively. Then, according to Equation (11), the absolute difference between the corresponding elements of the comparison sequence and the reference sequence of each evaluated object is calculated one by one. The minimum value is recorded as Δ(min), and the maximum difference is recorded as Δ(max). Finally, the data in the absolute difference are transformed by Equation (12) to obtain the correlation coefficient (ξ_0*i*_(*k*)). In the formula, (*ρ*) is the resolution coefficient, which is often set to 0.5.

(9) $$x_{i}\left(k\right)=\frac{x_{i}^{\prime }\left(k\right)-\underset{k}{\min}x_{i}^{\prime }\left(k\right)}{\underset{k}{\max}x_{i}^{\prime }\left(k\right)-\underset{k}{\min}x_{i}^{\prime }\left(k\right)}$$

(10) $$x_{0}\left(k\right)=\frac{x_{0}^{\prime }\left(k\right)-\underset{k}{\min}x_{0}^{\prime }\left(k\right)}{\underset{k}{\max}x_{0}^{\prime }\left(k\right)-\underset{k}{\min}x_{0}^{\prime }\left(k\right)}$$

(11) $$\Delta_{0i}\left(k\right)=\left|x_{0}\left(k\right)-x_{i}(k)\right|$$

(12) $$\xi_{0i}\left(k\right)=\frac{\Delta \left(\min\right)+\rho \Delta (\max)}{\Delta_{0i}\left(k\right)+\rho \Delta (\max)}$$

The correlation coefficients and grey relational degree are shown in Table 2 and Table 3. (ξ_0_) represents the correlation coefficient, and the grey relational degree is the average value of the (ξ_0_).

Analysis of the results in Table 2 and Table 3 reveals that the grey relational degree between the UCS and the corresponding CSH-MMT micro-interfacial energy is 0.8 for a varying water-to-cement ratio at a constant cement-to-soil ratio and is 0.69 for a varying cement-to-soil ratio at a constant water-to-cement ratio. The closer the grey relational degree is to 1, the better the correlation between the comparative sequence and the reference sequence is. Therefore, the results in Table 2 and Table 3 demonstrate that the energy in the interface binding region between CSH and MMT is an important factor influencing macroscopic performance. At the same time, it can be seen that adjusting the cement-to-soil ratio and water-to-cement ratio to maximize repulsion or minimize attraction at the CSH-MMT interface can enhance the stability of cement-stabilized montmorillonite soil and subsequently improve its mechanical performance. This is primarily due to the high repulsion energy at the interface effectively suppressing the dispersion of MMT. MMT, due to lattice substitution, attracts cations to its surface, which then diffuses into the solution in a specific sequence, creating a diffusion double layer [29,30] around it. When there are many cations in the system, the thickness of the double electric layer between the MMT layers increases, causing MMT to disperse. Greater repulsion at the CSH-MMT interface leads to a stronger inhibitory effect on the dispersion of MMT, resulting in greater system stability. Conversely, high attractive forces at the interface promote the dispersion of MMT. This increases the interlayer spacing of MMT and expands the lattice, leading to reduced system stability.

## 4. Discussion

Although grey relational analysis demonstrates a good correlation between the CSH-MMT interfacial energy and UCS of cement-stabilized montmorillonite soil, they are not entirely consistent. One reason for this disparity is that it is impossible to match the mass ratio of CSH, MMT, and water in macroscopic experiments with the model used in the MD simulations. Another reason is that the MD model only considers CSH and does not account for the influence of other hydration products of cement on interfacial energy. For example, as cement is an alkaline additive, its hydration results in the generation of a small amount of OH^−^. The H^+^ in the hydroxyl group on the edges of MMT tend to dissociate under alkaline conditions, and the higher the OH^−^ concentration, the greater the effect. This leads to a larger effective negative charge on the MMT layers, thus having a greater impact on the interfacial energy. Additionally, from a microscopic perspective, the pozzolanic reaction of MMT also influences the UCS of cemented soil.

**Figure 8:**
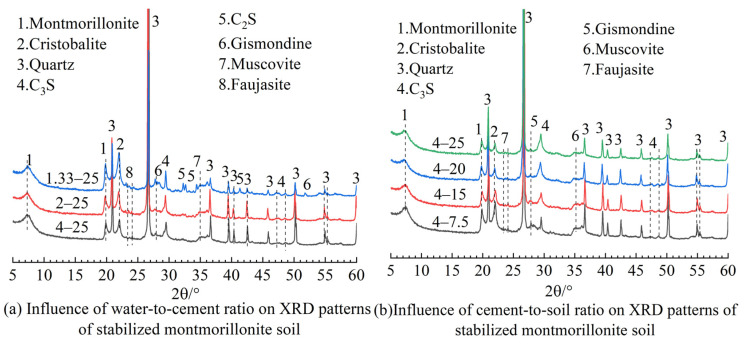
XRD spectra for cement-stabilized montmorillonite soil under different mix proportions.

In this regard, this section uses the XRD characterization method to further explore the reasons why the UCS of cemented soils and CSH-MMT interfacial energy are not completely consistent, and further validate the simulation results, ensuring the correctness of the MD model construction. Figure 8 presents the XRD spectra for cement-stabilized montmorillonite soil under different mix proportions.

Figure 8a demonstrates the influence of the varying water-to-cement ratio on the XRD reflection characteristics of cement-stabilized montmorillonite soil. The chemical reaction types in the cemented soil system mainly consist of cement hydration reactions and pozzolanic reactions with clay minerals, which are reflected in the XRD spectra as the diffraction peaks of cement hydration products and pozzolanic reaction products. The XRD spectra have diffraction peaks between 23 and 25° and 27 and 29°. After excluding the main crystalline reaction products, portlandite, etc., produced by cement hydration, it is determined that the diffraction peak in this range is generated from the common reaction products in clay minerals stimulated by alkalis—gismondine and faujasite [31,32]. The presence of these minerals is a part that cannot be reflected in molecular simulations, and these reaction products may have solid solubility with CSH, making it difficult to introduce them into the MD model, leading to differences between the simulation results and the evolution trend of strength. Nevertheless, the XRD reflection intensity of gismondine in the XRD spectra of samples 1.33–25 is higher than that of other samples. This reflects that, under low moisture content conditions, CSH undergoes a decalcification process, which is consistent with the conclusion in the molecular simulation process that calcium ions in the CSH layer migrate toward the MMT layer as water molecules decrease.

Figure 8b illustrates the influence of the cement-to-soil ratio on the XRD reflection characteristics of cement-stabilized montmorillonite soil under a water-to-cement ratio of 4. The test results show that the types of reaction products in cemented soil are generally consistent across samples with a different water-to-cement ratio. The impact mode of the cement-to-soil ratio on the reflection characteristics of cemented soil is relatively simple. As the cement-to-soil ratio increases from 7.5% to 25%, the intensity of the continuous reflection caused by reaction products such as gismondine and faujasite gradually decreases. This indicates that when there is a higher content of MMT, more Ca^2+^ and Na^+^ are involved in the chemical reaction process, which aligns with the observation in the molecular simulation process that the migration of Ca^2+^ and Na^+^ intensifies with an increasing number of MMT layers.

Therefore, although the MD method cannot fully simulate the physicochemical mechanisms of cemented soil, the XRD analysis results suggest that the ionic and molecular properties in the interface binding region between CSH and MMT correspond to actual conditions. This further validates the correctness of the MD model construction.

## 5. Conclusions

The current research on the kinetics of cemented soil performance development lacks an analysis of the binding mechanism at the nanoscale interface between cement hydration products and clay minerals, as well as an analysis of the energy source at the cemented soil interface. To address this, the present paper first reveals the interaction mechanism in the binding interface region between CSH and MMT layers from the perspective of the interfacial energy, using MD simulation technology. The interfacial energy of the CSH-MMT is composed of van der Waals forces, hydrogen bonding energy, and electrostatic interactions. The migration of cations and the content of water molecules at the interface are key factors affecting the strength of the hydrogen bonding network and the electrostatic interactions at the interface.

Subsequently, the role mechanism of the CSH-MMT interfacial energy on the macroscopic properties of cement-stabilized montmorillonite soil is discussed, and the feasibility of using MD simulation technology to decipher the mechanical effects of cemented soil is determined through grey relational analysis. This provides a new theoretical approach for effectively regulating the performance of cemented soil. According to the results of the grey relational analysis, the mechanism of the CSH-MMT interfacial energy changes, as revealed through the MD simulations at the atomic level, is highly consistent with the mechanical action mechanisms obtained through macroscopic experiments. Therefore, it is possible to optimize the mechanical properties of cemented soil by adjusting the water-to-cement ratio and cement-to-soil ratio to maximize repulsion or minimize attraction at the CSH-MMT interface. The specific reason for this is that when the interfacial energy exhibits high repulsion, the CSH-MMT interface inhibits the dispersion of MMT, resulting in a higher system stability and, consequently, improved mechanical performance.

Finally, the correctness and sources of deviation in the MD model construction were explored through XRD analysis. Changes in the content of reaction products such as gismondine and faujasite indirectly reflect the migration characteristics of Ca^2+^ and Na^+^ under the influence of a varying water-to-cement ratio and cement-to-soil ratio. In molecular simulations, these changes are represented by variations in the interfacial energy, subsequently impacting the macroscopic properties of cemented soil. Furthermore, these reaction products may be mutually soluble with CSH, which complicates their incorporation into the MD model, leading to discrepancies between the simulation results and strength evolution trends.

## Figures and Tables

**Table 1:** Physical properties of the cement.

Density/g cm^−3^	Specific Surface Area/m^2^ kg^−1^	Water Requirement of Normal Consistency/%	Initial Setting Time/min	Final Setting Time/min
3.03	397	27.5	195	305

**Table 2:** Correlation coefficient under the same cement-to-soil ratio and different water-to-cement ratio.

Water-to-Cement Ratio	1.33	2	3	4	Grey Relational Degree
ξ_0_	1	0.85	0.33	1	0.80

**Table 3:** Correlation coefficient under the same water-to-cement ratio and different cement-to-soil ratio.

Cement-to-Soil Ratio/%	7.5	15	20	25	Grey Relational Degree
ξ_0_	0.61	0.33	1	0.79	0.69

## Data Availability

Data are contained within the article.
